# Investigation of Nitrogen Removal in Flue Gas Desulfurization and Denitrification Wastewater Utilizing Halophilic Activated Sludge

**DOI:** 10.3390/toxics12100742

**Published:** 2024-10-13

**Authors:** Min Ren, Yuqi Wang, Huining Zhang, Yan Li, Keying Sun

**Affiliations:** 1 Marine Environmental Monitoring Centre of Ningbo, Ningbo 315100, China; renmin@ecs.mnr.gov.cn; 2 School of Civil Engineering, NingboTech University, Ningbo 315100, China; zjwangyuqi@163.com (Y.W.); liyanliyan@nbt.edu.cn (Y.L.); 22212280@zju.edu.cn (K.S.); 3 College of Civil Engineering and Architecture, Zhejiang University, Ningbo 315100, China

**Keywords:** flue gas desulfurization and denitrification wastewater, high sulfate, microbial community structure, nitrogen removal, synchronous nitrification and denitrification

## Abstract

In the process of flue gas desulfurization and denitrification, the generation of high-sulfate wastewater containing nitrogen is a significant challenge for biological wastewater treatment. In this study, halophilic activated sludge was inoculated in a Sequencing Batch Reactor to remove nitrogen from wastewater with a high sulfate concentration (60 g/L). With the influent concentration of 180 mg/L, the removal rate of total nitrogen was more than 96.7%. The effluent ammonium nitrogen concentration was lower than 1.94 mg/L, and the effluent nitrate nitrogen and nitrite nitrogen concentrations were even lower than 0.77 mg/L. The salt tolerance of activated sludge is mainly related to the increase in the content of ectoine in microbial cells. The Specific Nitrite Oxidation Rate is quite low, while the Specific Nitrite Reduction Rate and Specific Nitrate Reduction Rate are relatively strong. In the system, there are various nitrogen metabolic processes, including aerobic nitrification, anaerobic denitrification, and simultaneous nitrification–denitrification processes. By analyzing the nitrogen metabolic mechanisms and microbial community structure of the reaction system, dominate bacteria can be identified, such as *Azoarcus*, *Thauera*, and *Halomonas*, which have significant nitrogen removal capabilities.

## 1. Introduction

Flue gas desulfurization and denitrification generates a large amount of wastewater with high concentrations of sulfate salt and ammonia nitrogen, which has the characteristic of poor biodegradability. This makes biological treatment quite challenging, and hence, it has always been a difficulty in the field of wastewater treatment.

High salinity in wastewater can cause cell fluid separation in microorganisms, reducing the activity of metabolic enzymes, destroying the structure of enzymes, thereby inhibiting the growth and metabolism of microorganisms, affecting the removal of pollutants in wastewater by biological methods, and even causing the collapse of the reaction system [1,2]. Some high-salinity wastewater biological denitrification processes, such as membrane bioreactor (MBR) [3], biofilm reactors [4], and upflow anaerobic sludge bed (UASB) reactors [5], are quite complex. Ji et al. achieved a 95% nitrogen removal rate in simulated wastewater containing 60 g/L sulfate and 70 mg/L ammonium nitrogen (NH_4_^+^-N) by enriching halophilic microorganisms in the anoxic/oxic biofilm reactors [6]. The sequencing batch reactor activated sludge process (SBR) is a commonly used microbial treatment process, known for its simple construction and flexible operation. Utilizing the alternating aerobic and anaerobic conditions of SBR to domesticate halophilic activated sludge and enrich halophilic microorganisms capable of denitrification offers potential advantages for treating high-sulfate wastewater. Ji et al. acclimated heterotrophic nitrification and aerobic denitrification (HNAD) sludge, and achieved a nitrogen removal rate of more than 88% in 20 g/L NaCl-containing wastewater in SBR reactor by controlling the DO of 6 mg/L [7]. Huang et al. acclimated STAB sludge in three sequential batch reactors (SBR) with different NaCl salinities (50, 75, and 100 g/L), and the total nitrogen (TN) removal rates were 80.47%, 73.15%, and 65.53%, respectively [8].

However, the use of microbial methods to treat sulfate wastewater can easily lead to the proliferation of sulfate-reducing bacteria, resulting in the generation of toxic and harmful sulfides. Sulfur cycling and the production of sulfides were observed when using a modified sequencing batch biofilm reactor to treat high-salinity mustard tuber wastewater [9]. In practical industrial wastewater treatment, if these sulfides are produced, they not only inhibit microbial denitrification [10], but also lead to increased treatment costs. An anoxic–oxic–oxic (AOO) three-stage biofilter technology was used to treat wastewater containing 20 g/L sulfate generated by catalytic cracking units for desulfurization and denitrification, achieving a 94.8% removal rate for nitrate nitrogen (NO_3_^−^-N) and a 70% removal rate for TN [11]. However, there has been little research conducted on the biological denitrification of high-sulfate (60 g/L) flue gas desulfurization and denitrification wastewater. The success of biological denitrification under high-sulfate conditions often lies in the rapid acclimation of salt-tolerant bacteria and the creation of a suitable microenvironment that promotes their enrichment and stability. This study aims to provide a new solution for the practical engineering treatment of wastewater.

This study used gradient salt-tolerant halophilic activated sludge in an SBR to treat high-sulfate real wastewater from thermal power plant, verifying the feasibility of this method for denitrification of high-sulfate wastewater. The nitrogen metabolism mechanisms and microbial community structures in the reactor was conducted to explore the denitrification mechanism.

## 2. Materials and Methods

### 2.1. Characteristics of the Inoculated Activated Sludge and Real Wastewater

#### 2.1.1. Inoculated Activated Sludge

The halophilic activated sludge used in this study was obtained by salt-tolerant domestication of the ordinary sewage plant freshwater activated sludge, enabling it to survive in high-sulfate simulated wastewater at 60 g/L and possess efficient denitrification capabilities. To promote the retention of salt-tolerant bacteria in the sludge and eliminate autotrophic nitrification bacteria, high-salt wastewater was first introduced, and freshwater was then introduced in the reactor. The salt-tolerant sludge was enriched and gradually exhibited the capacity for simultaneous heterotrophic nitrification and aerobic denitrification (SND) [7].

#### 2.1.2. Real Wastewater Quality

The real wastewater from flue gas desulfurization and denitrification used in this study came from Sinopec Zhenhai Refining and Chemical Company, with water quality parameters shown in Table 1. NaHCO_3_ was added at 500 mg/L during the experiment to adjust the pH value.

### 2.2. Experimental Apparatus and Conditions

In this experiment, the Sequencing Batch Reactor (SBR) activated sludge process was employed. The reactor is an uncovered cuboid with dimensions of 200 mm × 200 mm × 300 mm, and the effective volume of the reaction zone is 8 L with dimensions of 200 mm × 200 mm × 200 mm.

The reactor’s cycle period is 12 h, with 4 L of water intake and discharge per cycle. The reaction system was aerated for 5 h, followed by an anaerobic phase for 6 h. After the anaerobic phase, the system settled for 30 min, then discharged and filled for 30 min. During the aeration phase, the dissolved oxygen (DO) level remained at 6–7 mg/L.

The experiment was divided into three stages: Stage I (Days 1–12) used simulated wastewater (60 g/L Na_2_SO_4_, 80 mg[COD]/L CH_3_COONa, 60 mg/L NH_4_^+^-N, 20 mg/L NO_3_^−^-N, 500 mg/L NaHCO_3_), and additional CH_3_COONa was added at the start of the anoxic phase at 320 mg[COD]/L; Phase II (12–24 days) used actual wastewater, and an additional 320 mg[COD]/L of CH_3_COONa was added at the start of the anoxic phase; Phase III (24–36 days) used actual wastewater, and an additional 640 mg[COD]/L of CH_3_COONa was added at the start of the anoxic phase.

### 2.3. Experimental Methods

#### 2.3.1. Analysis of Conventional Water Quality Indicators

Influent and effluent water samples of the reactor were collected every day. After centrifuging the water samples at 4000 r/min for 5 min, the supernatant was filtered through a 0.22 μm aqueous phase filter (polyether sulfone) and then stored in a refrigerator at 4 °C. The water quality indicators tested included NH_4_^+^-N, NO_3_^−^-N, NO_2_^−^-N, TN, and COD. The measurements were conducted in accordance with the standard methods outlined by the American Public Health Association [12].

The method used for the determination of hydrogen sulfide gas is listed below. The mud water was loaded into a 500 mL threaded gas washing bottle and placed in a constant-temperature magnetic stirrer bath. Pass the produced gases, including any possible H_2_S, through an excess of 1 mol/L NaOH solution to check for the presence of sulfides in the gases. During the aeration phase, gas was directly captured by NaOH solution every hour. At the end of the anoxic phase, a certain amount of N_2_ was introduced into the bottle, and all the gas was collected through the NaOH solution. The concentration of sulfides was analyzed using the methylene blue spectrophotometric method.

The nitrogen removal efficiency at various salinity stages within the reaction system can be further investigated by monitoring the nitrogen concentration changes over a unit of time and per unit of MLVSS. This involves assessing the Specific Ammonia Oxidation Rate (SAOR), Specific Nitrite Oxidation Rate (SNOR), Specific Nitrite Reduction Rate (SIRR), and Specific Nitrate Reduction Rate (SNRR), with all rates expressed in units of milligrams per gram of VSS per hour (mg/g[VSS]·h).

For SAOR and SNOR measurements: 1 L of activated sludge was mixed only with NH_4_Cl and NaNO_2_ in a beaker and stirred in a constant-temperature water bath at 25 °C under DO of 6–7 mg/L. Then, 500 mg/L NaHCO_3_ was introduced in the beaker and the concentrations of NH_4_^+^-N and NO_2_^−^-N were measured. Fitting curves were created with time on the horizontal axis and the concentrations of NH_4_^+^-N and NO_2_^−^-N shown on the vertical axis; the absolute values of the slopes represent SAOR and SNOR.

For SNRR and SIRR measurements: An amount of 1 L of activated sludge in a beaker was stirred in a constant-temperature water bath at 25 °C without aeration. NaNO_3_, NaNO_2_, and organic carbon source (sodium acetate) were then added in the beaker to reach a final C/N ratio of 10. The concentrations of NO_3_^−^-N and NO_2_^−^-N were also measured. Fitting curves was created with time on the horizontal axis and the concentrations of NO_3_^−^-N and NO_2_^−^-N were shown on the vertical axis. The absolute values of the slopes represent the values for SNRR and SIRR.

The determination of intracellular ectoine employs high-performance liquid chromatography.

#### 2.3.2. Study of Nitrogen Metabolism Mechanism

After the anoxic phase ended on day 36, 1 L of sludge water was taken for a beaker test. After settling the 1 L of sludge water for 30 min, 500 mL of the supernatant was removed, and 500 mL of real wastewater was added. The mixture was stirred in a constant-temperature water bath at 25 °C with DO of 6–7 mg/L. Every hour (half an hour after the start of the aeration and anoxic phases), 2 mL of sludge water was taken from the beaker with a centrifuge tube, with three parallel samples each time. After centrifuging the sludge water samples at 12,000 r/min for 5 min, the supernatant was collected and filtered, and then the concentrations of NH_4_^+^-N, NO_3_^−^-N, and NO_2_^−^-N were measured to plot a nitrogen transformation diagram.

#### 2.3.3. High-Throughput Sequencing

On the 36th day, a sludge sample was taken from the reactor, after appropriate pretreatment, sent to Shanghai Sangon Biotech for high-throughput sequencing. The detection method involved DNA extraction from the samples, followed by Polymerase Chain Reaction (PCR) amplification of the V4 region of the 16S rRNA gene. The entire process included (1) using a DNA extraction kit to extract DNA from the samples; (2) analyzing the quality, purity, and concentration of the extracted DNA; (3) amplifying the extracted DNA through PCR; and (4) conducting high-throughput sequencing on the Illumina platform at Shanghai Sangon Biotech.

## 3. Results and Discussion

### 3.1. Changes In Organic Matter Concentration in the Reactor

**Figure 1:**
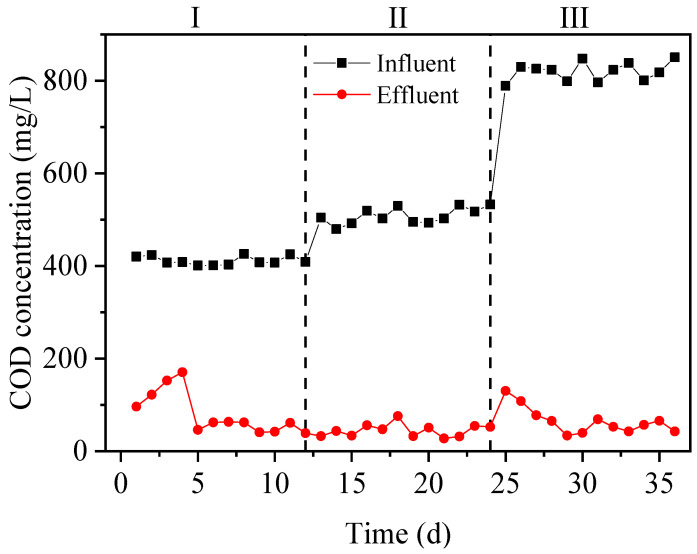
Change in COD in the reaction system. Stage I (Days 1−12): Simulated wastewater with 320 mg[COD]/L CH_3_COONa added at anoxic start. Phase II (Days 12−24): Actual wastewater with 320 mg[COD]/L CH_3_COONa at anoxic start. Phase III (Days 24−36): Actual wastewater only.

As shown in Figure 1, the graph represents the changes in COD in the reactor. In the first phase, the influent was simulated wastewater with a COD number of about 400 mg/L. The effluent COD was relatively high during the first four days and stabilized below 65 mg/L after the fifth day. In the second phase, 320 mg[COD]/L carbon source was added in the real desulfurization and denitrification wastewater during the anoxic stage, with a total influent COD of about 500 mg/L. The effluent COD during the second phase was below 76 mg/L. In the third phase, the influent was also using real desulfurization and denitrification wastewater, but the added carbon source during the anoxic stage was doubled to 640 mg[COD]/L, bringing the total influent COD number to around 800 mg/L. Due to the high nitrogen concentration in the real wastewater, more carbon sources were needed for cell metabolism of bacteria. When the carbon source was increased, the effluent COD number was temporarily increased on the 25th day, and then rapidly declined from the 26th day onwards and remained below 70 mg/L.

It is worth noting that sulfides were not detected throughout the experiment.

### 3.2. Nitrogen Removal Efficiency

**Figure 2:**
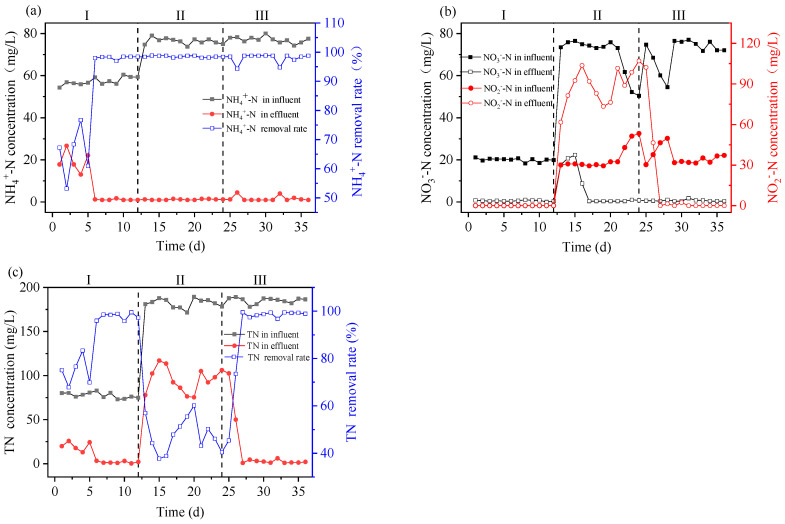
Changes in (**a**) NH_4_^+^-N, (**b**) NO_3_^−^-N and NO_2_^−^-N, and (**c**) TN concentrations and removal rates in the system.

The changes in NH_4_^+^-N concentration and removal rate within the reaction system are shown in Figure 2a. In the first phase, as the activated sludge had just entered a new reactor, the effluent NH_4_^+^-N concentration was high for the first five days, with the lowest removal rate being only 53.2%. Starting from the sixth day, the activated sludge gradually adapted to the conditions of the reaction system, and the NH_4_^+^-N removal rate rapidly increased, remaining above 97.0%. In the second and third phases, aside from fluctuations on the 26th and 32nd days, the NH_4_^+^-N removal rate in the reactor consistently stayed above 97.4%. This surpasses the results of other studies. For instance, in a 2.6% salinity condition, the ammonia nitrogen removal rate exceeds 82% [13]. This indicates that the reaction system has a strong nitrification capacity for NH_4_^+^-N in actual desulfurization and denitrification wastewater.

The changes in NO_3_^−^-N and NO_2_^−^-N concentrations within the reaction system are shown in Figure 2b. In the first phase, the effluent concentrations of NO_3_^−^-N and NO_2_^−^-N were 0.37 mg/L and 0 mg/L, respectively, indicating that the activated sludge had a significant removal efficiency on NO_X_^−^-N. In the second phase, the influent concentrations of NO_3_^−^-N and NO_2_^−^-N increased by using real wastewater. Between the 13th and 16th days, due to the influent total nitrogen (TN) concentration approaching 180 mg/L, the influent NO_3_^−^-N and NO_2_^−^-N concentrations reached as high as 76.50 mg/L and 38.90 mg/L, respectively. The effluent NO_3_^−^-N and NO_2_^−^-N concentrations peaked at 22.29 mg/L and 103.66 mg/L, respectively. After the 17th day, as the reactor gradually adapted to the high-nitrogen real wastewater, the effluent NO_3_^−^-N concentration quickly decreased and remained at 0.37 mg/L. However, the effluent NO_2_^−^-N concentration fluctuated between 73.38 and 106.97 mg/L and was much higher than the influent, suggesting incomplete denitrification due to insufficient carbon sources during the anoxic stage. Therefore, on the 25th day, the added carbon source in the reactor was doubled to 640 mg[COD]/L, and it was evident that the effluent NO_2_^−^-N concentration rapidly decreased between the 25th and 26th days, and from the 31st day onwards, the effluent NO_2_^−^-N concentration remained at 0 mg/L.

The changes in total nitrogen (TN) concentration and removal rate within the reaction system are shown in Figure 2c. In the first phase, due to changes in influent water and operating conditions, the high effluent NH_4_^+^-N concentration between day 1 and day 5 resulted in a lower TN removal rate. However, as the NH_4_^+^-N removal rate increased, starting from the sixth day, the TN removal rate remained above 95.8%. In the second phase, with actual desulfurization and denitrification wastewater as the influent and a C/N ratio of about 2.67, the carbon source was insufficient, leading to high effluent NO_X_^−^-N concentrations and a maximum TN removal rate of only 60.2%. In the third phase, with the doubling of the added carbon source, the C/N ratio was about 4.44, and from the 27th day onwards, the TN removal rate remained above 96.7%, with the effluent TN concentration below 6.10 mg/L, effectively removing most of the nitrogen from the real wastewater. This indicates that with a C/N ratio of about 4.44, the activated sludge has a good removal efficiency on nitrogen from actual desulfurization and denitrification wastewater.

### 3.3. Analysis of Law of Nitrogen Transformation and the Activated Sludge Characteristics

**Figure 3:**
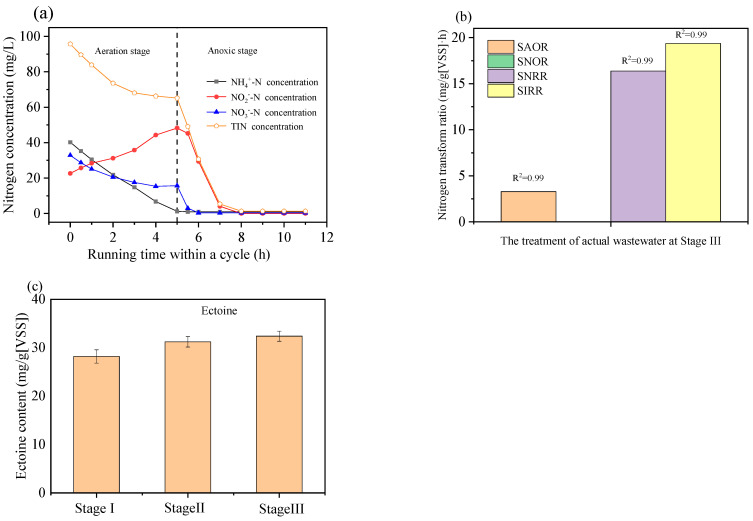
Changes in nitrogen transformations and the activated sludge characteristics: (**a**) Change in nitrogen concentrations in one cycle of the system at the end of Stage III; (**b**) SAOR, SNOR, SNRR, and SIRR in the treatment of real wastewater at Stage III; (**c**) Intracellular ectoine contents of activated sludges.

As shown in Figure 3a, the nitrogen concentration in the reaction system at the end of Phase III were depicted over one cycle. As the aeration time extended, the concentrations of NH_4_^+^-N, NO_3_^−^-N, and the total inorganic nitrogen (TIN) declined. This suggests that simultaneous nitrification and denitrification (SND) occurred within the reaction system during the aeration phase. Notably, at the 4th hour of aeration, the concentrations of NO_3_^−^-N and TIN cease to decrease, whereas the concentration of NH_4_^+^-N continues to drop. This could be due to the scarcity of organic carbon sources in the real wastewater. As the aeration progresses, these carbon sources are gradually depleted, insufficient for sustaining SND reactions [14]. However, autotrophic nitrification can still occur. Therefore, it can be observed from the figure that aerobic nitrification, anaerobic denitrification, and SND were all present in this reaction system, contributing to the reaction and removal of nitrogen from the wastewater.

Figure 3b illustrates the SAOR, SNOR, SNRR, and SIRR during the third phase of treating real wastewater. The values for SAOR, SNOR, SNRR, and SIRR were, respectively, 3.29, 0, 16.37, and 19.36 mg/g[VSS]. Compared to the nitrogen conversion rates at a salinity of 60 g/L during the acclimation phase, the SNOR remained at 0, indicating that nitrite oxidizing bacteria (NOB) indeed could not survive under high-salinity conditions. However, there is an increase in SAOR, SNRR, and SIRR, which may be due to changes in the denitrifying bacteria during the real wastewater treatment phase. Additionally, the enhanced denitrification could be attributed to the higher amount of external carbon source added during the anoxic phase. Ge et al. [15] found that as salinity increases, the activity of NOB decreases, while the activity of ammonia-oxidizing bacteria (AOB) first decreases but then rises again after a period of acclimatization due to AOB’s ability to produce ammonia monooxygenase to counteract salinity. Additionally, Gao et al. [16] discovered that with rising salinity, certain NOB species like Nitrobacter are directly eliminated at a salinity level of 15 g/L, while other NOB species like *Nitrospira*, although still present in saline water, have their activity greatly suppressed, leading to an accumulation of NO_2_^−^-N in the reaction system.

Figure 3c shows the changes in the intracellular ectoine content at different stages of treating real wastewater. The contents of ectoine in Stages I, II, and III were 27.52, 31.79, and 32.38 mg/g[VSS], respectively. Compared to the acclimation Phase III under a 60 g/L salinity reaction system, the content of intracellular ectoine in Stage I decreased when dealing with real wastewater. With the real wastewater being used as influent, the content of ectoine increased in Stage II, but only slightly increased in Stage III when the external carbon source was doubled. Ectoine was identified as a compatible solute synthesized by halophilic microorganisms, which can mitigate the detrimental effects of high salinity on proteins, biological membranes, and even the integrity of entire cells [17]. Wang et al. [18] found that *Halomonas* synthesizes ectoine concentrations of 150 and 420 mg/L under conditions of 30 g/L NaCl and 60 g/L NaCl, respectively. Studies have shown that the production of ectoine is greatly related to the concentration and type of carbon and nitrogen sources [19]. Therefore, the decrease in the intracellular ectoine content in stage I might be due to the depletion of the external carbon source, while the increase in Stages II and III could be attributed to the rise in the total nitrogen from the external carbon source and the wastewater. Overall, the content of intracellular ectoine during both the acclimation phase under 60 g/L salinity and the treatment of real wastewater was significantly higher than that in low-salinity systems, further confirming that the content of ectoine is a key factor for microbial salt tolerance.

### 3.4. Microbial Community Structure Analysis

**Figure 4:**
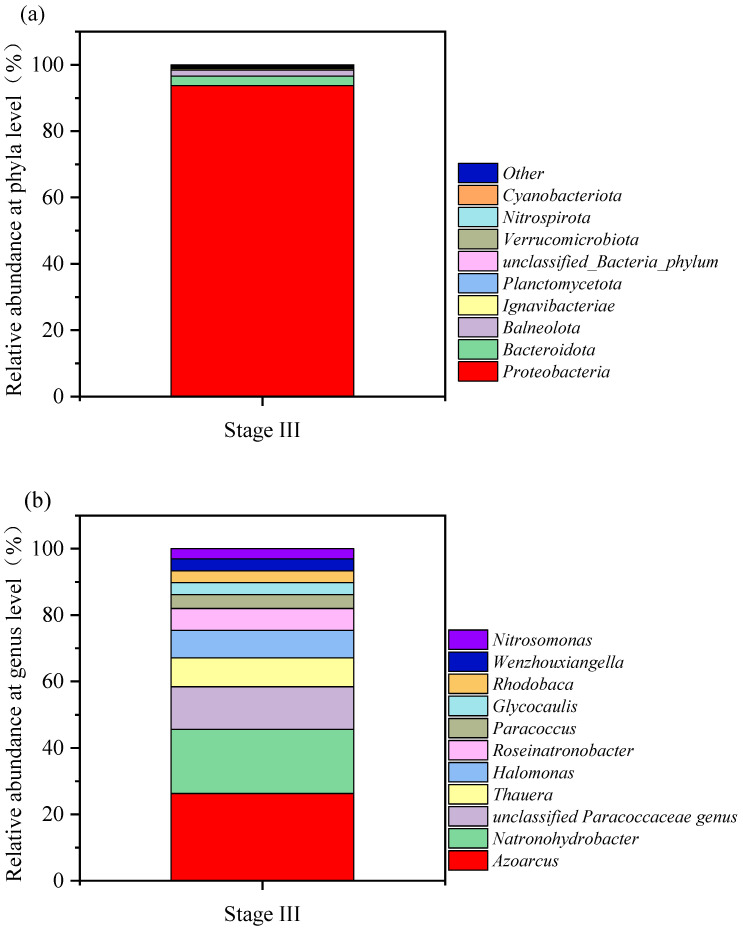
Relative abundance of Stage III microorganisms at phyla level (**a**) and genus level (**b**).

Figure 4 illustrated the relative abundances of microbial communities at the phylum level (a) and genus level (b) during Phase III. As shown in Figure (a), Proteobacteria was the dominant phylum, accounting for as much as 93.72%. Figure (b) further reveals genera with higher abundances, including *Azoarcus* (18.38%), *Natronohydrobacter* (13.51%), unclassified *Paracoccaceae* genus (8.97%), *Thauera* (6.06%), *Halomonas* (5.80%), *Roseinatronobacter* (4.61%), *Paracoccus* (2.91%), *Glycocaulis* (2.53%), *Rhodobaca* (2.51%), *Wenzhouxiangella* (2.48%), and *Nitrosomonas* (2.17%). Notably, *Azoarcus*, *Thauera*, *Paracoccus*, *Halomonas*, and *Nitrosomonas* were all typical denitrifying bacteria. References [20,21] confirmed the denitrification capabilities of *Azoarcus* and *Paracoccus*, while *Nitrosomonas* [22] could perform nitrification in high-salinity wastewater. In addition, *Thauera* [23] and *Halomonas* [24] not only had the abilities for nitrification and denitrification but could also carry out the simultaneous nitrification and denitrification (SND) process.

Among the most common identified species, *Azoarcus taiwanensis* and *Natronohydrobacter thiooxidans* stood out. *Azoarcus taiwanensis* was a facultative denitrifying and sulfite-oxidizing bacterium [25], while *Natronohydrobacter thiooxidans* was a heterotrophic sulfite-oxidizing bacterium [26]. This finding indicated that the real wastewater might contain a certain amount of sulfite. In addition to sulfur and nitrogen compounds, flue gas desulfurization and denitrification wastewater generated by petrochemical plants might also contain some recalcitrant organic substances, such as toluene and phenol. Relevant studies have shown that *Azoarcus* [27], *Natronohydrobacter* [28], *Roseinatronobacter* [29], *Paracoccus* [21], and *Wenzhouxiangella* [30] could utilize these recalcitrant organic substances as carbon sources in saline wastewater. It was worth noting that *Roseinatronobacter* also had the ability to oxidize sulfur-containing compounds such as sodium thiosulfate [29,31].

## 4. Conclusions

In the denitrification experiment of actual wastewater from thermal power plants, using halophilic activated sludge achieved significant results:

(1) Under the condition of a C/N ratio of 4.4, the TN removal rate could reach 96.7%, and the concentrations of NH_4_^+^-N, NO_3_^−^N, and NO_2_^−^-N in the effluent were all below 1.94 mg/L, 0.77 mg/L, and 0.77 mg/L, respectively, providing an effective method for the treatment of desulfurization and denitrification wastewater.
(2) The salt tolerance of activated sludge was related to the increase in the content of ectoine in microbial cells. Under high-salinity conditions, the denitrification process was significant, while the nitrification process was weak, and various nitrogen metabolic processes occur within the system.
(3) An analysis of the system revealed the nitrogen removal capabilities of dominant bacteria species such as *Azoarcus*, *Thauera*, and *Halomonas*, which played a key role in the community.

## Figures and Tables

**Table 1:** Quality index of real wastewater from desulfurization and denitrification.

Na_2_SO_4_ (g/L)	COD (mg/L)	NH_4_^+^-N (mg/L)	NO_3_^−^-N (mg/L)	NO_2_^−^-N (mg/L)	pH
60	80–200	70–80	60–80	30–50	7–9

Notes: chemical oxygen demand is defined as COD; nitrite nitrogen is defined as NO_2_^−^-N.

## Data Availability

The data presented in this study are available in this article.
